# Down-regulation LncRNA-SNHG15 contributes to proliferation and invasion of bladder cancer cells

**DOI:** 10.1186/s12894-021-00852-1

**Published:** 2021-05-20

**Authors:** Aldhabi Mokhtar, Chuize Kong, Zhe Zhang, Yan Du

**Affiliations:** Department of Urology, The First Hospital of China Medical University, 155 Nanjing North Street, Shenyang, Liaoning 110001 P.R. China

**Keywords:** Long noncoding RNA, SNHG15, Cell invasion, Cell proliferation, Bladder cancer

## Abstract

**Objectives:**

The aim of this study was to investigate the effect of lncRNA-SNHG15 in bladder carcinoma using cell lines experiments and the relationship between clinical characteristics and lncRNA-SNHG15 expression was analyzed.

**Methods:**

Bladder cancer tissues and near-cancer tissues were collected. The real-time PCR (RT-PCR) was used to detect the expression of lncRNA-SNHG15 in tissues and cell lines. The expression of lncRNA-SNHG15 was downregulated by interference (siRNA), as detected by RT-PCR, that was used to determine the efficiency of the interference. CCK-8 and Transwell assays were used to evaluate the effect of lncRNA-SNHG15 on the proliferation and invasion capability of bladder cancer cells. The t-test was used for Statistical analyses, which were carried out using the Statistical Graph pad 8.0.1.224 software.

**Result:**

The expression of lncRNA-SNHG15 was up regulated in 5637, UMUC3 and T24 cell lines compared with corresponding normal controls (*P*<0.05). Up regulation was positively related to tumor stage (*P*=0.015). And tumor size (*P*=0.0465). The down-regulation of lncRNA-SNHG15 with siRNA significantly inhibited UMUC3 and T24 cell proliferation and invasion.

**Conclusion:**

This study showed that lncRNA-SNHG15 is overexpressed in bladder cancer tissues and (5637, UMUC3 T24) cell lines. Up regulation was positively related to tumor stage (*P*=0.015), and tumor size (*P*=0.0465). Down-regulation of lncRNA-SNHG15 by siRNA significantly inhibited UMUC3 and T24 cell proliferation and invasion, indicating a potential molecular target for future tumor targeted therapy.

## Background

Bladder cancer have a higher incidence in urinary malignancies. Because of its susceptibility to recurrence, progression, and metastasis, the current ideal treatment for bladder cancer is a comprehensive including surgery treatment, chemotherapy and Radiation therapy, but the overall effect is limited, and the invasion and metastasis are the main reasons of bladder cancer treatment failure [1]. Primary bladder tumors usually arise from the muscularis and mucosal epithelium, with the latter accounting for approximately 95% of cases, which about 9095% are urothelial carcinoma. Bladder cancer has the characteristics of Polycentricity and recurrence over time, as well as the biological characteristics of local invasion and metastasis and a high postoperative recurrence rate. 7080% of bladder cancer patients are initially diagnosed with noninvasive urinary epithelial papilloma. After active surgery and bladder perfusion treatment, 50% of patients are still diagnosed with non-invasive urinary papilloma. 70% of the patients relapse within 5years, and 1030% of patients develop invasive urothelial cancer [2]. Following the sequencing of the human genome, it was found that the proportion of protein-coding genes in the entire human genome sequence was less than 3%, and that more than 80% of the sequences were frequently transcribed into RNA without protein-coding functions. Non-coding RNA is RNA that does not have protein-coding functions. Non-coding RNA is actually a complex network of gene expression regulation that plays a key role in regulating many important biological functions of cancer cells. It is classified into two types based on the length of the sequence: short non-coding RNA and long non-coding RNA. Long non-coding RNA (lncRNA) is a class of transcripts with more than 200 nucleotides, and those without protein-coding functions are mostly transcribed by polymerase II [3]. Although lncRNA cannot be translated into protein, it has effects on life activities such as gene transcription regulation, proteins post-translational modification, and epigenetic regulation of gene expression. It is closely related to pathophysiological changes, disease diagnosis and treatment [4]. LncRNA-SNHG15, an intergenic lncRNA found on chromosome 7p13, belongs to a non-coding class of RNAs that includes snoRNAs [5]. Increasingly, studies show that LncRNA-SNHG15 has abnormally expression in many types of tumors, such as renal cancer [6], lung cancer [7], colorectal carcinoma [8], prostate cancer [9]. Long non-coding RNASNHG 15 promotes cell proliferation in glioma microvascular endothelial cells [10], and also contributes to osteosarcoma cell proliferation, invasion and autophagy [11]. However, the expression and function of lncRNA-SNHG15 in bladder cancer is ambiguous. The aim of this study was to investigate the effect of lncRNA-SNHG15 in bladder carcinoma using cell lines experiments and the relationship between clinical characteristics and lncRNA-SNHG15 expression was analyzed. We investigated the role of lncRNA-SNHG15 in bladder carcinoma using data and cells line PCR, cck-8, and other experimental techniques to detect the expression of lncRNA-SNHG15 in bladder cancer tissues and cells line. The results showed that overexpression of lncRNA-SNHG15 was an associated molecular change in bladder carcinoma tissues, and cell lines (5637, UMUC3, T24). As a result, the effects of aberrant lncRNA-SNHG15 expression on the biological behavior of UMUC3 and T24 cell lines were also investigated. The results provided novel insights into the function and mechanisms of lncRNA-SNHG15 bladder carcinoma pathogenesis, and lncRNA-SNHG15 was identified as a potential therapeutic target for cancer intervention.

## Methods

### Cell lines

The human bladder cancer cell lines (5637, UMUC3, J82, T24) and the normal bladder epithelial SVHUC1 cell line (SV-HUC-1) were obtained from the Chinese Academy of Sciences, Type Culture Collection Cell Bank (Shanghai, China). The cells (SV-HUC-1) were cultured in Hams F12 medium (Sigma, St. Louis, MO, USA). Fetal bovine serum (FBS),RNA extraction reagent Trizol and reverse transcription kit were purchased from japan (TaKaRa Co. Tokyo Japan), lncRNA-SNHG15interference RNA (siRNA lncRNA-SNHG15), and si-NC were obtained from Shanghai Jima Co(Shanghai, China), CCK-8 reagent were purchased from the Invitrogen Corporation (Carlsbad, CA, USA), Transwell chamber from the BD Biosciences (Franklin Lakes, NJ, USA).

### Tissue samples

Between March and September 2018, 30 patients were diagnosed with bladder cancer and underwent surgical resection at the First Affiliated Hospital of China Medical University (Shenyang, China). Surgical bladder cancer tissues and matched near-cancer tissues were collected. The Ethics Committee on Human Research of China Medical Universitys First Affiliated Hospital approved the current study, and all patients provided written informed consent. Tissue samples were collected and stored at 80C before being used.

### Real-time PCR

(1) Design the primer sequence as follows:

- Reverse primer: ACCTGTACTCCGTACTCCGT.
- Forward primer: GGCGGTGGATGACTAGACTG.

In addition, we use the Takara fluorescence quantitative PCR kit to set up 10 reaction systems using the TRIZOL method. Fill a 96-well plate with the above 10-L reaction system. Each sample had three auxiliary. When determining the expression level of LncRNA-snhg15, GAPDH was used as an inner. On a real-time quantitative PCR instrument, the green dye method was used to assess the expression of LncRNA-snhg15 in bladder cancer tissues and matched adjacent tissues, with GAPDH serving as an internal reference. Each sample was run at least three times, and the average CT value was calculated and analyzed.

### Cell transfection assay

Cells were cultured in RPMI1640 medium containing, 10% FBS, placed in 5% CO2 incubator at 37C. When the cells were in the logarithmic growth phase, at a density of about 40% in 6-well plates. Each well received 5L Lipofectamine 2000 and 200pmol siRNA lncRNA-SNHG15 mixture.

The siRNA sequence forLncRNA-SNHG15 was: GGAUUUAAAUAUGUGAAAA.

### Cell proliferation by cck-8 assay

Cells were plant seeds in 96-well plates at a thickness 210^3^ cells/well were transfected with SNHG15 NC siRNAs for 72h in situ. Cell proliferation was evaluated using Cell Counting Kit-8 (CCK-8) assay (Dojindo Molecular Technologies, Inc., Shanghai, China) conforming to the manufacturer's protocol. The decadic was projected at 450nm each 24h utilizing a plate reader (Model 680; Bio-Rad Laboratories, Inc., Watford, UK).

### Cell invasion with transwell assay

A Transwell chambers with a8-m matrix gel coating hole is inserted into the 24 hole plate. Transwell kit without matrix gel was used for invasive test. After transfection of snhg15 and NC siRNAs for 72h, the cells were trypsinized and suspended in RPMI-1640 containing 1% FBS. Subsequently, 0.2ml of cell suspension (1104/ml) was added to the upper cavity and 0.6ml of RPMI-1640 containing FBS was added to the lower cavity. After incubation at 37 for 24h, the remaining cells in the upper cavity were removed. The cells moving to the lower side were fixed with 4% paraformaldehyde for 10min and stained with 1.0% crystal violet at room temperature for 10min. The image was taken with EVOS XL core imaging system (Invitrogen; Thermo Fisher Scientific, Inc.).

### Statistics

Statistical analyses were performed using the Statistical Graph pad 8.0.1.224 software, and the t-test was used.

## Results

### The expressions of lncRNA-SNHG15 in bladder cancer tissues and its relationship to clinical features

**Fig. 1:**
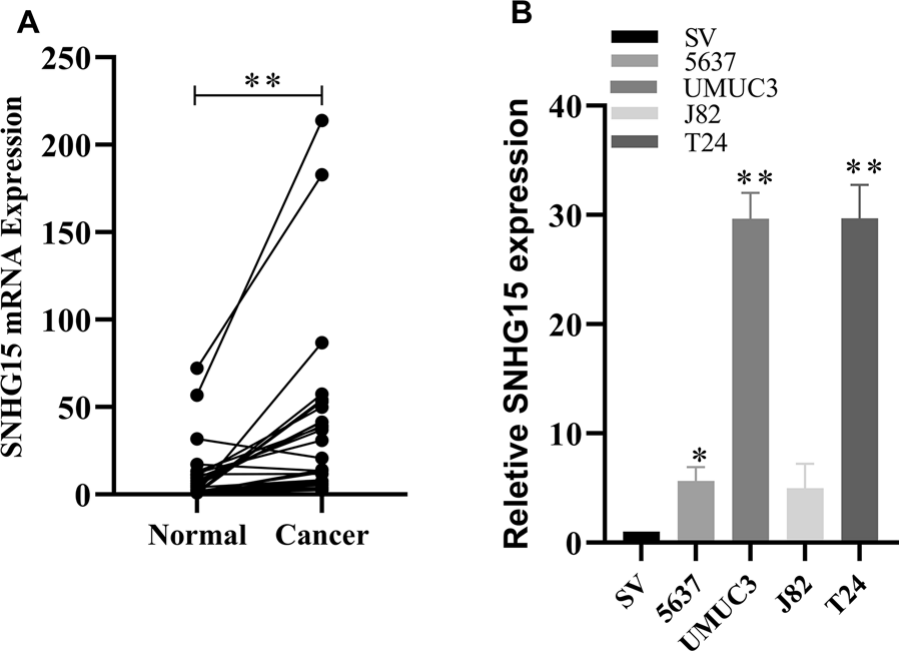
Using the paired t test, *P<0.05, LncRNA-SNHG15 Expression was found to be higher in bladder cancer tissues. LncRNA-SNHG15 Was up regulated in the bladder cancer cell lines. By RT-PCR, the relative Expression of LncRNA-SNHG15 was detected in tumor cells lines (5637, UMUC3, T24)and compared with the normal cell line (SV-HUC1) by RT-PCR *P<(0.05)

Interestingly, qRT-PCR results revealed that lncRNA-SNHG15 expression was higher in bladder cancer tissues than in adjacent tissues (*p* 0.05, Fig.1a). The expression of lncRNA-SNHG15 is associated with clinical and pathological features. We investigated the relationship between increased lncRNA-SNHG15 expression levels and clinical characteristics in 30 bladder cancer cases to see if lncRNA-SNHG15 expression is related to clinical features. LncRNA-SNHG15 up-regulation was positively correlated with tumor stage (*P*=0.015) and tumor size (*P*=0.0465). However, lncRNA-SNHG15 expression levels were not related to other factors such as patient age, gender, tumor number, or nodal invasion. (See Table 1) This finding also suggested that lncRNA-SNHG15 was an important molecule in the development of bladder cancer.

**Table 1:** The relationship between the expression of lncrna-snhg15 and clinical statistics

Factor	No	LncRNA-SNHG15		*p*
		High	low	
Total	30	19	11	
Gender				
Male	24	15	9	0.999
Female	6	4	2	
Age (year)				
50	25	17	8	0.3268
<50	5	2	3	
Tumor size (cm)				
3	19	15	4	*0.0465
<3	11	4	7	
Tumor stage				
Ta & T1	9	16	4	*0.015
T2	21	3	7	

The expression level of LncRNA-SNHG15 was related to tumor size (*P*=*0.0465), degree of immersion (*P*=*0.015), metastases, but not related to gender and age

### The effect of lncRNA-SNHG15 on bladder cancer cell proliferation, invasion and metastasis

**Fig. 2:**
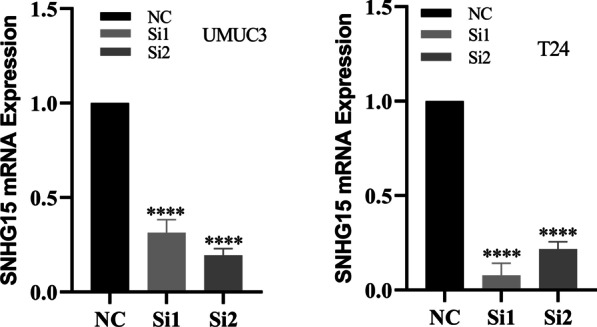
(T24, UMUC3) Down regulation of LncRNA-SNHG15bysi RNA*P<0.05, compared to B with si-NC

**Fig. 3:**
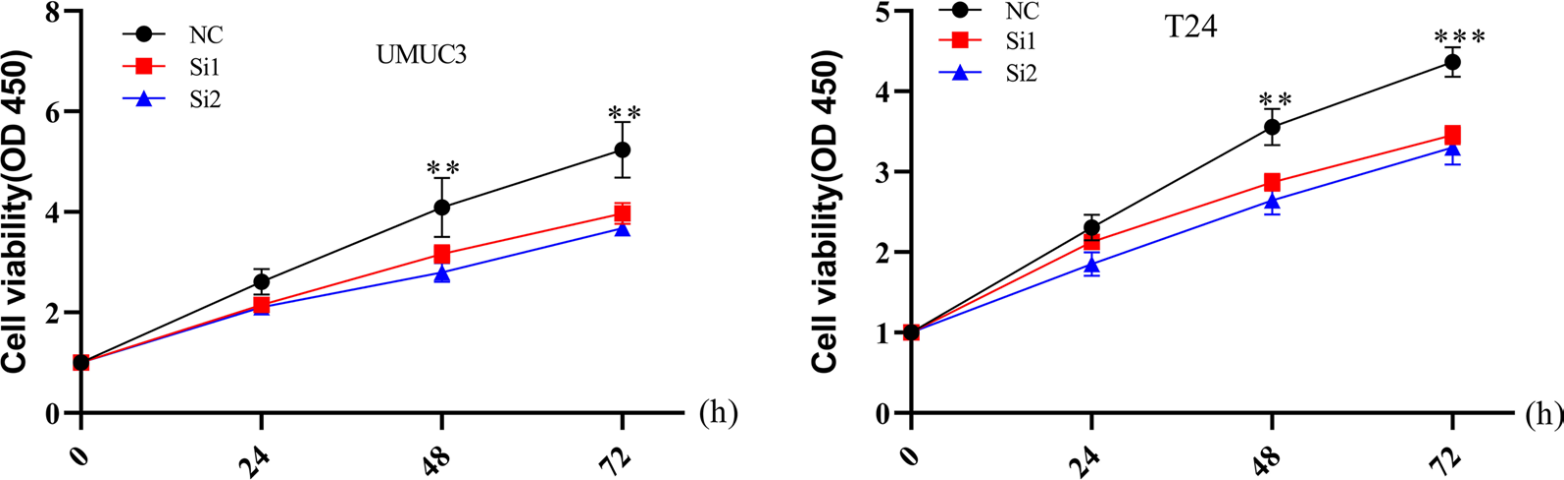
TheCCK-8 assay was used performed to detect cell proliferation differences between si-NC and si-LncRNA-SNHG15*P<0.05, when compared with si-NC

**Fig. 4:**
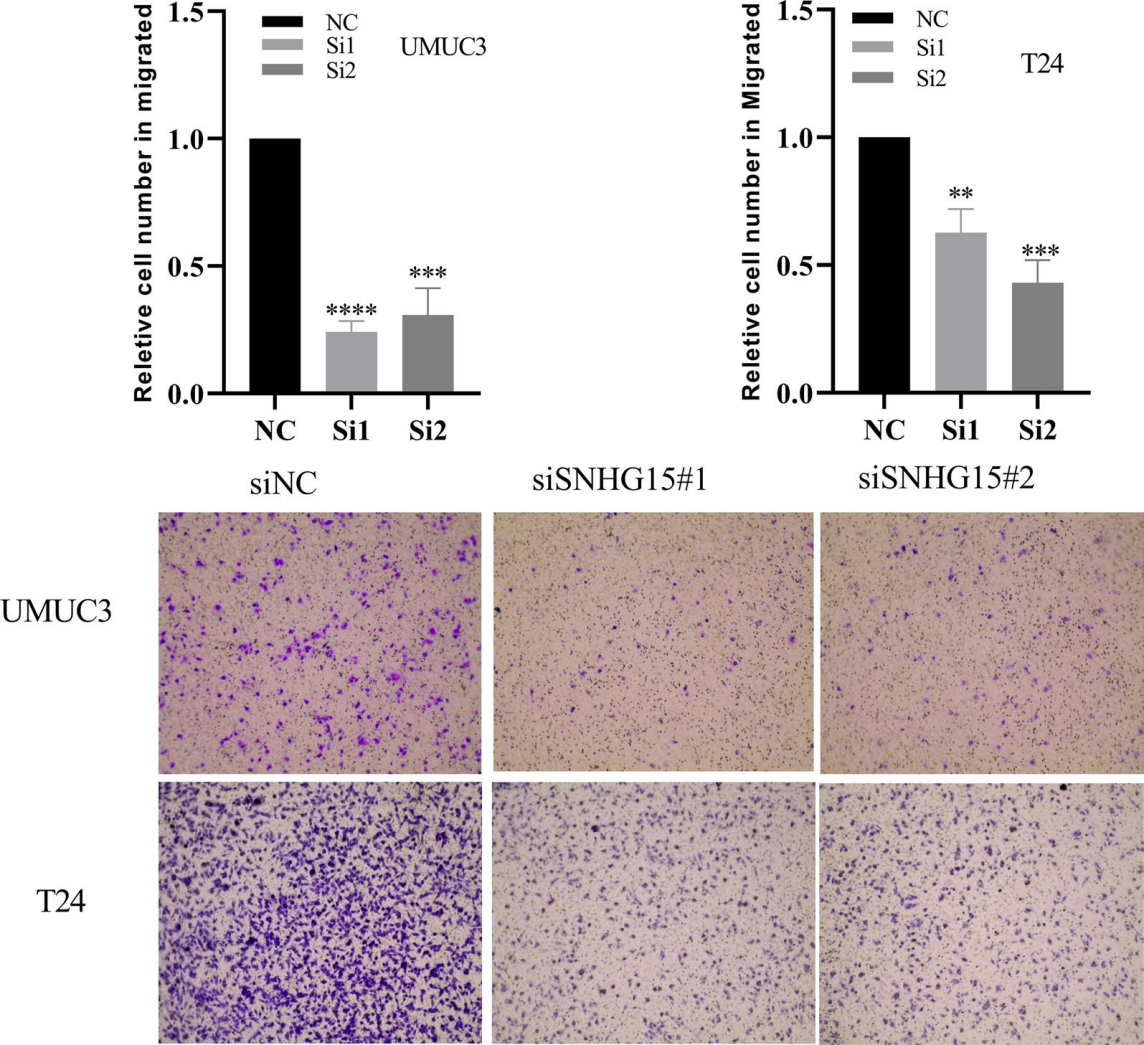
Down-regulating the expression of LncRNA-SNHG15 can significantly inhibit the invasiveness of bladder cancer cells

The results of qRT-PCR showed that the expression of lncRNA-SNHG15 in bladder cancer (5637, UMUC3, T24) cell lines was higher than that in normal bladder epithelial (SV-HUC-1) cell line (*p*<0.05; Fig.1b). To investigate the function of lncRNA-SNHG15, a siRNA targeting lncRNA-SNHG15 was transfected into UMUC3 and T24 cells. RT-qPCR revealed that lncRNA-SNHG15 was significantly downregulated 48h after transfection of siRNA in the bladder cancer UMUC3 and T24 cell lines compared to the control group (*p*<0.05; Fig.2). **CCK-8 assays** showed that siRNA lncRNA-SNHG15 could inhibit the proliferation of lncRNA-SNHG15 in bladder cancer bladder cancer UMUC3 and T24 cell lines (*p*<0.05; Fig.3). In addition, a Transwell invasion assay was also used to examine the effect of lncRNA-SNHG15 on the migration capacity of UMUC3 and T24 cells. According to the Transwell invasion assay, down-regulation of lncRNA-SNHG15 expression significantly inhibits the invasion of bladder cancer (UMUC3 and T24) cell lines (*p*<0.05, Fig.4).

## Discussion

According to Antoni et al. reported that an estimated 430.000 new cases of bladder cancer were diagnosed in 2012, making bladder cancer the ninth most common cancer worldwide [12]. The 5-year recurrence-free survival rate for highly localized invasive bladder cancer after a radical cystectomy ranges between 62 and 89% [13]. Emerging evidence have suggests that lncRNAs act as oncogenic or suppressor genes in a variety of cancers, regulating transcription and post-transcription [14]. The underlying mechanism that regulates development of bladder cancer is largely unknown. The development of novel molecular biomarkers for the diagnosis and prognosis of bladder cancer is an urgent requirement. Long non-coding RNAs (long ncRNAs, lncRNA) are a type of RNA, defined as being transcripts with lengths exceeding 200 nucleotides that are not translated into protein. It is considered a byproduct of RNA transcription, and even "noise" in the transcription process of human genes, because it does not participate in protein coding and has no biological functions [15]. However, lately research found that LncRNA participates in the regulation of multiple signal pathways in cells through chromosome modification, transcription, and interference. Our team Du [6], has found that LncRNA-SNHG15 is highly expressed in renal cancer tissues, which is closely related to the proliferation, apoptosis, invasion and migration of renal cancer. The present study explored the expression of LncRNA-SNHG15 in bladder urothelial carcinoma using clinical characteristics in the 30 bladder cancer cases to determine whether and cell experiments. The results demonstrated that the expression was apparently localized in the adjacent tissues. The results show that the high expression of LncRNA-SNHG15, its effect the tumor malignant, more aggressive, strong invasion ability, and the possibility of recurrence after the surgery. In order to study the potential effect of LncRNA-SNHG15 on bladder cancer cell lines, In vitro cell experiments in this study showed that the expression of LncRNA-SNHG15 in bladder cancer cells was significantly up-regulated in bladder cancer (5637, UMUC3,T24) cell lines. Transfection of specific si-RNA LncRNA-SNHG15 can inhibit the expression of LncRNA-SNHG15 in bladder cancer UMUC3, T24 cell lines. The data from the present study implied that LncRNA-SNHG15 distributed in the nucleus may serve its role by regulating the expression level of si-RNA. However, the underlying mechanisms require additional investigation. In summary, the present study demonstrated that LncRNA-SNHG15 has potential as a clinically promising biomarker for bladder urothelial carcinoma. LncRNA-SNHG15 regulated the proliferation and migration of 5637, UMUC3 and T24 cells, and these data may provide novel insights into molecular cancer therapy. We propose the following additional research on in vitro binding experiments: the p65 recombinant protein and snhg15 were co-incubated in vitro for an in vitro binding experiment to investigate the p65 functional region binding to snhg15.

## Conclusion

In summary, this is the first study to investigate the lncRNA-SNHG15 in bladder carcinoma. This study discovered that lncRNASNHG15 is overexpressed in bladder cancer tissues and cells. Down regulation of lncRNA-SNHG15 can inhibit bladder cancer cell proliferation and invasion, making it a potential molecular target for future tumor targeted therapy. Certainly, additional potential functions and mechanisms must be investigated.

## Data Availability

The datasets used and/or analyzed during the current study are available from the corresponding author on reasonable request.

## Abbreviations

FBS: fetal bovine serum
LncRNA: long non-coding RNA
miRNA: microRNA
PBS: phosphate-buffered saline
RT-PCR: real-time PCR
SNHG15: small nucleolar RNA host gene 15
Bcc: bladder cell
RPMI-1640: Roswell Park Memorial Institute
cck-8 assay: cell counting kit-8-cell proliferation and cytotoxicity assay
NC: normal cell
